# Distinct Assembly Mechanisms Underlie Similar Biogeographic Patterns of Rare and Abundant Bacterioplankton in Cascade Reservoirs of a Large River

**DOI:** 10.3389/fmicb.2020.00158

**Published:** 2020-02-07

**Authors:** Juan Chen, Peifang Wang, Chao Wang, Xun Wang, Lingzhan Miao, Sheng Liu, Qiusheng Yuan, Shenghao Sun

**Affiliations:** Key Laboratory of Integrated Regulation and Resource Department on Shallow Lakes, Ministry of Education, College of Environment, Hohai University, Nanjing, China

**Keywords:** bacterioplankton community, biogeographic pattern, cascade dams, rare taxa, distance-decay relationship

## Abstract

Bacterioplankton communities commonly consist of few highly abundant species and a large number of rare species that play key roles in biogeochemical cycles of aquatic ecosystems. However, little is known about the biogeographic assemblies of these communities, especially in large rivers suffering from cascade dam regulation. Here, we used a *16S rRNA* gene amplicon sequencing approach to investigate the biogeographic patterns and underlying assembly mechanisms of abundant and rare bacterioplankton taxa in cascade reservoirs of the Jinsha River in China. The results revealed species loss of bacterioplankton due to dam construction, which was more significant for rare taxa than for abundant ones. The distributions of abundant and rare taxa exhibited similar spatial and temporal patterns, which were significantly distinct between winter and summer and between upstream and downstream reservoirs. Both spatial (dispersal-related process) and environmental (selection process) factors seemed to together govern the assembly and biogeography of abundant and rare taxa, although both factors explained only a small fraction of variation in the rare taxa. More importantly, environmental factors explained more community variation in abundant sub-community than that in rare sub-community. Co-occurrence network analysis revealed that abundant species with closer interactions were more often located in a central position of the network compared with rare species. Nevertheless, half of the keystone species were rare species and may play important roles in maintaining the network stability. Overall, these findings indicate that distinct assembly mechanisms underlie the similar biogeography of rare and abundant bacteria in cascade reservoirs of a large river.

## Introduction

Rivers serve as pipes that shuttle water, organic materials, and nutrients from continents to oceans, providing numerous essential ecosystem services (Read et al., 2015; Savio et al., 2015). Bacterioplankton plays vital roles in biogeochemical reactions, nutrient cycling, and as members of the food web, thus contributing greatly to maintenance of the function and stability of river ecosystems (Ruiz-Gonzalez et al., 2013). Aquatic bacterioplankton is highly diverse, with a few very abundant taxa and a large number of rare species with lower abundance (the “rare biosphere”) (Rappé et al., 2000; Sogin et al., 2006; Pedrós-Alió, 2012; Mo et al., 2018). Most previous studies have focused on the abundant taxa due to their important roles in carbon cycling and biomass production (Pedrós-Alió, 2012; Liu et al., 2015a). However, recent studies have emphasized the critical ecological functions of rare taxa, which include more metabolically active organisms than abundant taxa (Lynch and Neufeld, 2015; Wu et al., 2017). The loss of rare species in an ecosystem may induce serious ecological consequences (Pendleton et al., 2014). In the past few years, the rapid development of high-throughput sequencing facilitated a more comprehensive survey of rare bacterioplankton communities (Jiao et al., 2017; Xue et al., 2018). Recent studies have compared the composition and dynamics of abundant and rare bacterioplankton communities in sea waters (Lindh et al., 2015; Zhang et al., 2018), epipelagic waters (Wu et al., 2017), subtropical bays (Mo et al., 2018), and lakes (Liu et al., 2015a). However, there is a significant knowledge gap in our understanding of the biogeography of abundant and rare bacterioplankton in rivers.

Many large rivers worldwide are regulated by damming to meet growing energy or water supply demand (Nilsson et al., 2005). Dams, especially cascade hydropower dams, are serious anthropogenic disturbances that alter the natural flow regime and modify the transport of riverine sediments, nutrients, and biota (Timpe and Kaplan, 2017). The reservoir formed upstream of a dam changes the water environment from lotic to lentic, affects water quality, and disturbs nutrient transport along the river by retaining suspended particles and sediments (Li et al., 2012; Yan et al., 2015). The lentic environment of reservoir can act as a “species filter” to reduce the biodiversity of aquatic animal and phytoplankton species reliant on free-flowing water (Agostinho et al., 2004; Liu et al., 2015b). The significant physical and chemical changes of a waterbody due to dam construction can greatly affect the bacterioplankton community composition (Ruiz-Gonzalez et al., 2013; Wang et al., 2018) and may further impact the biogeochemical cycle and ecological function of river systems. Most previous studies considered the effects of a single dam on bacterioplankton by comparing the upstream and downstream sections of a reservoir (Yan et al., 2015; Wang et al., 2017). However, there are few studies of the biogeography patterns of abundant and rare taxa and their assembly mechanisms in cascade reservoirs along a large river, despite the fact that the “cumulative” ecological effects of multiple dams along a river are more significant than those caused by a single dam (Timpe and Kaplan, 2017).

Recent studies have increasingly emphasized the importance of dispersal limitation and environmental selection in microbial community assemblages (Logue and Lindström, 2010; Lindstrom and Langenheder, 2012; Lindh et al., 2016; Jiang et al., 2018). The community composition of bacterioplankton in aquatic ecosystems can be affected by various environmental parameters such as water temperature, dissolved organic matter, and dissolved silicate (Savio et al., 2015; Gao et al., 2017; Wang et al., 2018). Spatial factors such as longitude and latitude can also affect the distribution of bacterioplankton community because of species drift or dispersal limitation over spatial distance (Hayden and Beman, 2016; Liu et al., 2018). There is growing evidence that microbial biogeographic pattern is driven by both dispersal limitation and environmental selection; however, identifying the relative influence of these factors remains a central issue in microbial ecology. Some bacterial community studies have investigated the relative importance of the two processes (Hanson et al., 2012; Jiang et al., 2018). However, thus far, the predominant process that controls biogeography of abundant and rare bacterioplankton in rivers, particularly in cascade dam-affected rivers, has not been determined. Abundant and rare taxa may have different responses to environmental changes and different dispersal potential along spatial gradients (Magurran and Henderson, 2003; Yan et al., 2017). The dispersal rate of rare taxa would likely be lower than abundant taxa due to their lower abundance. In contrast, the most abundant bacteria can disperse readily, as many more individuals can potentially participate in a dispersal event.

Compared to the abiotic factors, much less is known about how species interactions may influence microbial communities. In natural habitats, tens of thousands of microbial species live together within complex ecological networks with various types of interactions (e.g., mutualism, competition, parasitism, and predation) (Faust and Raes, 2012). These biotic interactions can be depicted using a network model, in which each node represents an observed species and the edge connecting two nodes represents significant correlations between the two species (Deng et al., 2012; Hu et al., 2017). Co-occurrence network analysis has been increasingly used as a tool to unravel the intraspecies or interspecies interactions in complex microbial assemblages across a wide range of environments from the human gut (Greenblum et al., 2012) to soils (Ma et al., 2016; Xiao et al., 2018), oceans (Aylward et al., 2015), and lakes (Jones et al., 2018). Topological network properties (e.g., complexity and modularity) can indicate the critical role of species interactions in governing community assembly and ecosystem function (Cardona et al., 2016). In addition, some keystone species within a co-occurrence network may play critical roles in maintaining the structure and function of the microbial community (Ma et al., 2016; Xue et al., 2018). Most previous network analyses of bacterioplankton have focused on entire communities in oceans (Steele et al., 2011; Aylward et al., 2015) and lakes (Zhao et al., 2016). Until now, this approach to analyze the co-occurrence patterns of abundant and rare bacterioplankton has not been applied to cascade dam-affected rivers.

The Jinsha River is the upper reaches of the Yangtze River, has a total river length of 2,279 km and a catchment of 473,200 km^2^, and has attracted extensive attention as a significant hydropower resource (Wang et al., 2015a). According to China’s State Power Planning, a cascade of 24 hydroelectric dams is planned along the mainstream of the Jinsha River. Of these 24 dams, eight have already been constructed and are currently operational, forming huge cascade reservoirs and providing substantial hydropower. However, the large-scale construction of cascade dams can significantly alter the river hydrology, aquatic ecology, and local environment conditions, which might influence the microbial community habitat and ecosystem function in reservoirs. Several recent studies reported reduced diversity of bacterioplankton communities in the impoundments of cascade dams, with significant effects on taxonomic and functional compositions in large rivers like the Lancang River (Wang et al., 2018). Given the importance of abundant and rare taxa in maintaining the health of river ecosystems, it is urgent to investigate their variations in cascade reservoirs.

Here, we carried out high-throughput 16S *rRNA* gene sequencing to investigate the biogeographic assembly pattern of the overall community as well as abundant and rare bacterioplankton communities in water samples collected from cascade reservoirs of the Jinsha River. We specifically focused on the following questions: (i) If cascade dam construction affects the diversity and community composition of bacterioplankton? (ii) Are the effects of multiple dams cumulative or largely insignificant? (iii) Do abundant and rare bacterioplankton exhibit similar biogeographic patterns in the cascade reservoirs of the Jinsha River? (iv) What are the major factors influencing the assembly of the bacterioplankton community, and do the contributions of these factors differ for abundant and rare taxa? (v) What are the co-occurrence patterns of abundant and rare taxa, and which taxa occupy more important positions in the co-occurrence network?

## Materials and Methods

### Study Area and Sampling Collection

The Jinsha River located in the upper reaches of the Yangtze River is one of the largest rivers in southwestern China. The Jinsha River is strongly regulated by intensity hydropower development, and eight dams namely Liyuan (LY), Ahai (AH), Jinanqiao (JAQ), Longkaikou (LKK), Ludila (LDL), Guanyinyan (GYY), Xiluodu (XLD), and Xiangjiaba (XJB) have been constructed and form a cascade of eight reservoirs in the mainstream. Detailed information about these eight dams is shown in Supplementary Table S1. The field sampling was conducted in summer (May 2017) and winter (December 2017), and no extreme weather occurred during our sampling period. For each hydropower station, at least one site is in the reservoir area, one site is within 5 km before the dam site (dam-affected site), and one is within 5 km after the dam site (dam-controlled site). A total of 26 sites (Supplementary Figure S1) were sampled in the cascade reservoir area in each sampling time, and the sampling sites of the two sampling seasons are the same. At each sampling site, three water samples were collected with a Ruttner sampler (Hydro-Bios, Altenholz, Germany) from a water depth of approximately 50 cm and were then homogenized as one sample. Samples (2.0 L) for bacterioplankton analysis were filtered through 0.22-μm Millipore membranes (Millipore, MA, USA) by vacuum filtration and the membranes were stored at −80°C until DNA extraction.

During sampling, a multiparameter water quality analyzer (HQ40d, Hach, CO, USA) was used for *in situ* monitoring of dissolved oxygen (DO), pH, and water temperature. The turbidity was measured using a turbidimeter (2100 P, Hach, CO, USA). Other water chemistry parameters, including total nitrogen (TN), total phosphorus (TP), dissolved reactive silicon (Dsi), nitrate (NO_3_^−^), and total organic carbon (TOC) were measured in the laboratory as detailed by Wang et al. (2017). The site names, locations, and water chemical data are given in Supplementary Table S2.

### DNA Extraction and Bacterial 16S *rRNA* Gene Amplicon Sequencing

DNA was extracted from 0.22-μm membrane filters using the PowerWater DNA Extraction kit (MoBio, CA, USA) according to the manufacturer’s instructions. Then, the DNA was quantified using a Nanodrop 1000 spectrophotometer (Thermo Scientific, DE, USA). The universal primer pair 515F (5′-GTGTGCCAGCMGCCGCGGTAA-30) and 806R (5′-GGACTACHVGGGTWTCTAAT-3′), with a unique 6 bp barcode added to the 5′ terminus of 515F, was used to amplify the V4 region of bacterial *16S rRNA* genes. Genomic DNA was amplified in triplicate 30 μl PCR reactions containing 0.3 μM of forward and reverse primers, 15 μl of Phusion® High-Fidelity PCR Master Mix (NEB, Ipswich, MA, USA), and 10 ng genomic DNA using the thermal cycling procedure described previously (Zhang et al., 2017). PCR products were pooled together and purified using Agarose Gel DNA purification kit (TaKaRa). The PCR products from each sample were combined in equimolar ratio, and the obtained library was sequenced on the Illumina MiSeq PE250 platform. The sequencing data were submitted to NCBI Sequence Read Archive database under accession numbers SRP219313 and SRP219317.

### Sequencing Data Processing and Analysis

Raw Illumina paired-end reads were merged by FLASH1.2.7 (Magoc and Salzberg, 2011), and quality filtering of reads was conducted using the Quantitative Insights Into Microbial Ecology (QIIME) pipeline (Caporaso et al., 2010). Reads, which were shorter than 200 bp or had an average quality score less than 25, were discarded, and chimeras were picked out using USEARCH to obtain high quality sequences. The quality sequences with 97% similarity were assigned as the same operational taxonomic units (OTUs) by UCLUST (Edgar, 2010). The most abundance sequence from each OTU was selected as the representative sequence, and all representative sequences were aligned by PyNAST. Taxonomic identity of each OTU was predicted by the ribosomal database project (RDP) classifier using the Greengenes database^1^. To standardize sequencing effort across samples, the OTU table was rarefied down to 21,633 sequences per samples for data analyses, according to the minimal sequence depth among all samples (core_diversity_analyses.py, QIIME).

The abundant and rare taxa were defined based on relative abundance cut-offs (Liu et al., 2015a; Xue et al., 2018). To facilitate comparisons between studies, the selection of the cutoff points refers to previous studies on bacterioplankton (Liu et al., 2015a; Liao et al., 2017; Mo et al., 2018). In this study, locally abundant OTUs were defined as those with relative abundances ≥1% within a sample, and locally rare OTUs were defined as those with abundances <0.01% within a sample. The OTUs with a mean relative abundance of ≥0.1% in all samples were considered as regionally abundant taxa, whereas those with a mean relative abundance of <0.001% in all samples were defined as regionally rare taxa. The downstream analyses were performed at three levels including the total community, regionally abundant, and regionally rare taxa.

Alpha diversity indices (Species richness and Pielou’s evenness) of the total community, abundant, and rare taxa were calculated in R with the vegan package. Non-metric multidimensional scaling (NMDS) ordination was performed to visualize the dissimilarity of bacterioplankton communities among samples (beta diversity) based on the Bray-Curtis distance. The analysis of similarity (ANOSIM) was used to measure the significant differences on bacterioplankton communities between sampling seasons and between reservoir locations. To explore the impacts of environmental filtering, all water chemical parameters, with the exception of pH, were log (*x* + 1) transformed to improve homoscedasticity and normality (Mo et al., 2018). Mantel tests were used to identify which water chemical properties were significantly correlated with the bacterioplankton communities.

To determine the distance decay relationship, we analyzed the correlations between the Bray-Curtis similarity of bacterioplankton communities and spatial distance among sampling sites and the environmental distance based on Euclidean distance. The community similarity, spatial distance, and environmental distance matrixes were linearized using PASSAGE2 (www.passagesoftware.net). The distance decay curve was plotted by logarithmic community similarity against logarithmic spatial and environmental distance (Tian et al., 2018). A set of spatial variables (e.g., PCNM1, PCNM2, etc.) were calculated based on the longitude and latitude coordinates of each sampling site using principal coordinates of neighbor matrices (PCNM), a powerful tool that reflects the spatial scales of autocorrelation (Legendre et al., 2008). Variance partitioning analysis (VPA) was used to calculate the proportion of variance in the bacterioplankton community that could be explained by spatial distance (based on PCNM) and environmental variables. Before VPA, we conducted a forward selection procedure to select subsets of spatial and environmental variables. All the above statistical analyses were conducted in R3.2.1 (https://www.r-project.org/) with the “vegan” package.

To reduce the complexity of the data sets, only OTUs present in more than five samples with more than 10 sequences were retained for the network construction. A total of 3,489 OTUs were used for the network analyses. Spearman’s rank correlation was used to evaluate pairwise associations among those OTUs in R with “picante” package (Xue et al., 2018). Robust correlations were defined as those with Spearman’s correlation coefficients > 0.8 and *p* < 0.01 (Xue et al., 2018). Network-level topological properties (average degree, clustering coefficient, average path length, graph density, network diameter, and modularity) of the abundant and rare subcommunities and four node-level topological feature (degree, betweenness centrality, closeness centrality, and eigenvector centrality) were further calculated in the “igraph” R package. Network visualization was made with Gephi version 0.9.2. Nodes with high degree (> 20) and low betweenness centrality values (< 5,000) are recognized as keystone species (Ma et al., 2016).

## Results

### Alpha Diversity of the Bacterioplankton Community

Bacterioplankton was collected from 52 water samples and subjected to *16S rRNA* amplicon sequencing to yield a total of 2,269,162 high-quality reads, which clustered into 10,384 OTUs based on 97% similarity. At local levels, no OTU was always abundant (≥1% in all samples) and only two OTUs (133,118 sequences) with ≥1% abundance were present in >70% of the samples, while 7,133 OTUs (40,776 sequences) were always locally rare (<0.01% in all samples) (Supplementary Figure S2). The proportions of locally abundant and rare OTUs were relatively constant across all samples, with ranges of 0.38–2.94% for abundant OTUs and 59.3–82.7% for rare OTUs (Supplementary Table S3). At regional levels, the data included 153 (1.47%) OTUs with 1,731,879 (76.3%) sequences, which were classified as the abundant taxa, and 7,907 (76.1%) OTUs with 48,643 (2.14%) sequences, which were classified as rare taxa. The abundant taxa account for >50% relative abundance and <5% richness, and the rare taxa contribute <5% relative abundance and >50% richness, which exactly matched with other publications (Logares et al., 2014; Zhang et al., 2018).

**Figure 1:**
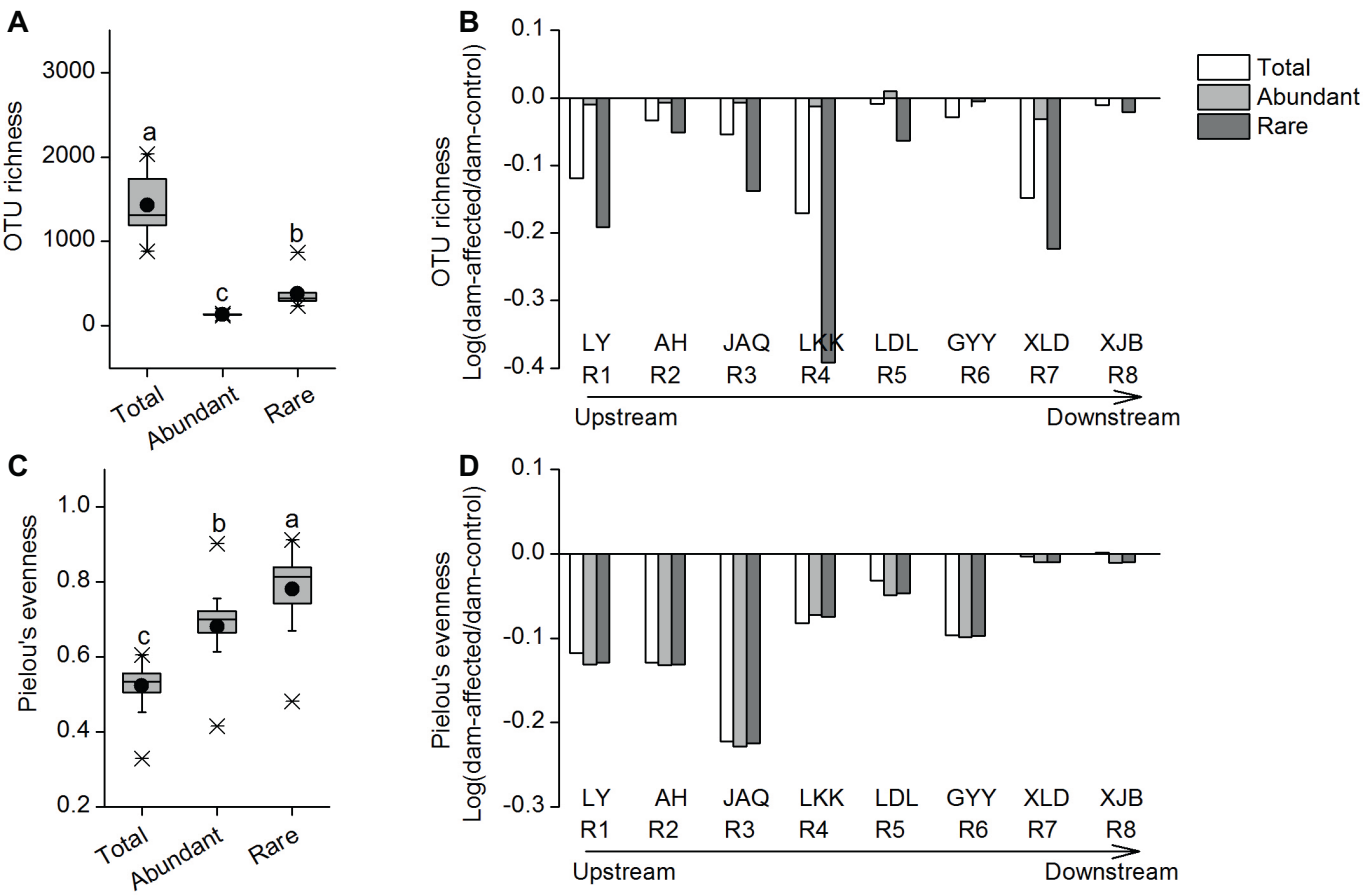
OTU richness **(A)** and Pielou’s evenness **(C)** of the total, abundant, and rare bacterial communities in the cascade reservoirs of the Jinsha River and variation of OTU richness **(B)** and Pielou’s evenness **(D)** in dam-controlled and dam-affected sites of each dam. In **(A)** and **(C)**, box is drawn to represent values from lower 1/4 quantile to upper 1/4 quantile, medians are drawn as black bars within boxes; boxes with different lowercase letters represent significant differences among the three taxa at *p* < 0.05 according to one-way ANOVA. In **(B)** and **(D)**, the columns show the logarithmic OTU richness and Pielou’s evenness ratios of the dam-affected site to those for the dam-controlled site corresponding to each dam. Positive values represent increased richness or evenness, and negative values represent decreased richness or evenness due to dam construction. R1: Liyuan (LY), R2: Ahai (AH), R3: Jinanqiao (JAQ), R4: Longkaikou (LKK), R5: Ludila (LDL), R6: Guanyinyan (GYY), R7: Xiluodu (XLD), R8: Xiangjiaba (XJB). Total: whole bacterioplankton communities; Abundant: abundant taxa; Rare: rare taxa.

The OTU richness of the total taxa varied from 885 to 2,034, that of the abundant taxa varied from 120 to 146, and that of the rare taxa varied from 238 to 866 (Figure 1A). The mean Pielou’s evenness values of the total, abundant, and rare taxa were 0.52, 0.68, and 0.78, respectively (Figure 1C). The rare taxa exhibited significantly higher alpha diversity, in terms of OTU richness and Pielou’s evenness, than the abundant taxa (Figures 1A,C). Among all sampling sites, OTU richness of the total and rare taxa was lowest in the LDL area (four sites from LDL.1 to LDL.C), and that of the abundant taxa exhibited a gradually increasing trend along the river; whereas Pielou’s evenness of the three groups of taxa showed no clear longitudinal pattern from the upstream to the downstream (Supplementary Figure S3). For most of the reservoirs, the OTU richness and Pielou’s evenness of the three taxa groups were lower in dam-affected site than in dam-controlled site (Supplementary Figure S3).

To further characterize the effect of dam-formed reservoirs on species diversity, we calculated the logarithmic OTU richness and Pielou’s evenness ratios of the dam-affected site to those for the dam-controlled site corresponding to each dam. Positive values represented increased richness or evenness, and negative values represented decreased richness or evenness due to dam construction. The changes of OTU richness and Pielou’s evenness of total, abundant, and rare taxa were generally negative in eight reservoirs, with an exception of abundant taxa for one reservoir (LDL), indicating that dam construction caused species loss of bacterioplankton in water. The effect of dam construction on OTU richness differed among different reservoirs, with the stronger effect on rare species in LKK and the weaker effect in GYY and XJB than other reservoirs. The rate of decrease in richness gradually increased from AH to LKK in the upstream reservoirs, whereas no clear pattern was observed in the downstream reservoirs from LDL to XJB (Figure 1B). The decreases in Pielou’s evenness were comparable for abundant and rare taxa, while there was a more significant decrease in OTU richness for the rare taxa than the abundant taxa (Figures 1B,D).

### Beta Diversity and Abundance-Occupancy Relationship

**Figure 2:**
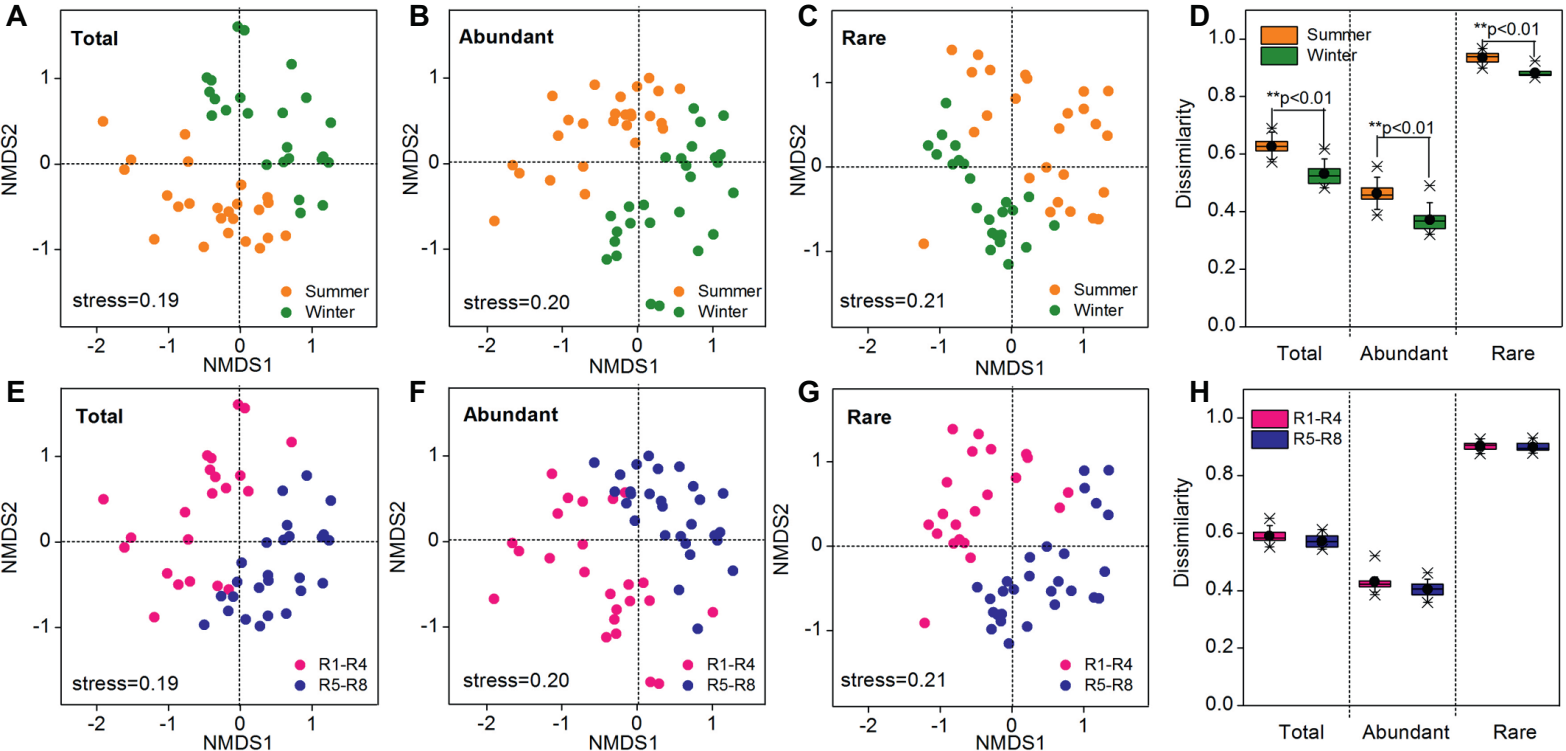
Bacterioplankton community composition of all 52 water samples as indicated by non-metric multidimensional scaling (NMDS) **(A–C, E–G)** and community dissimilarity of the total, abundant, and rare taxa in different sampling seasons **(D)** and locations **(H)**. Sampling plots in NMDS analysis have been color-coded according to sampling season (summer vs. winter) and reservoir location (R1-R4 vs. R5-R8). R1-R4 includes 12 sites in upstream reservoirs from Liyuan to Longkaikou, and R5-R8 includes 14 sites in downstream reservoirs from Ludila to Xiangjiaba in each sampling time. Total: whole bacterioplankton communities; Abundant: abundant taxa; Rare: rare taxa.

As revealed by NMDS, the community compositions of the total, abundant, and rare taxa in total samples demonstrated clear seasonal groups (Figures 2A–C). The samples from upstream reservoirs (R1-R4, 12 sites from LY.1 to LKK.C) and downstream reservoirs (R5-R8, 14 sites from LDL.1 to XJB.C) were grouped separately by NMDS (Figures 2E–G). The consistency of the results was confirmed by the ANOSIM statistic test, which showed significant differences in community composition between summer and winter (*p* < 0.01), and also between upstream and downstream reservoirs (*p* < 0.01), for the total, abundant, and rare taxa (Table 1). Beta diversity was estimated as average pairwise community dissimilarity within each sampling season (summer and winter) and within each reservoir location (R1-R4 and R5-R8). Pairwise community similarity was calculated based on OTUs by using Bray-Curtis metric. The rare taxa showed significantly higher beta diversity than the total community, and both had significantly higher diversity than that of the abundant taxa. The average community dissimilarity for the total, abundant, and rare taxa groups in summer samples was significantly higher than that in winter samples (*p* < 0.01), while the samples from different reservoir locations (upstream reservoir vs. downstream reservoir) did not significantly differ in their average community dissimilarity (*p* > 0.05) (Figures 2D,H).

**Table 1:** Analysis of similarities (ANOSIM) of the total, abundant, and rare bacterioplankton taxa between different sampling seasons (summer vs. winter) and between different reservoir locations (upstream vs. downstream) in the cascade reservoirs of the Jinsha River.

Pairwise tests	Total		Abundant		Rare	
	*r*	*p*	*r*	*p*	*r*	*p*
Summer vs. winter	0.453	0.001	0.438	0.001	0.446	0.001
Upstream vs. downstream	0.487	0.001	0.487	0.001	0.456	0.001

*The results are generated using the pairwise Bray-Curtis dissimilarity data. Total: whole bacterioplankton communities; Abundant: abundant taxa; Rare: rare taxa; *r*: correlation; *p*: significance*.

Venn diagram analysis showed that there were no shared OTUs between the summer rare OTUs and abundant OTUs in winter, and between the summer abundant OTUs and rare OTUs in winter (Supplementary Figure S4), indicating that the summer rare OTUs did not become abundant OTUs in winter and vice versa. The abundant OTUs were shared between the upstream and downstream reservoir sites, but the percentage of shared OTUs for rare taxa was only 57.0% (Supplementary Figure S4). The abundant OTUs (149 out of 153, 97.3%) occupied >50% of samples, while 34.8% of rare taxa (2,755 out of 7,907) was distributed in only one or two samples and none of them occupied >50% of samples (Supplementary Figure S4). Spearman’s rank correlation showed that the mean relative abundance of abundant taxa (*r* = 0.33, *p* < 0.001) was less positively correlated to the numbers of samples occupied compared to that for rare taxa (*r* = 0.83, *p* < 0.001) (Supplementary Figure S4).

### Taxonomic Composition of the Bacterioplankton Community

**Figure 3:**
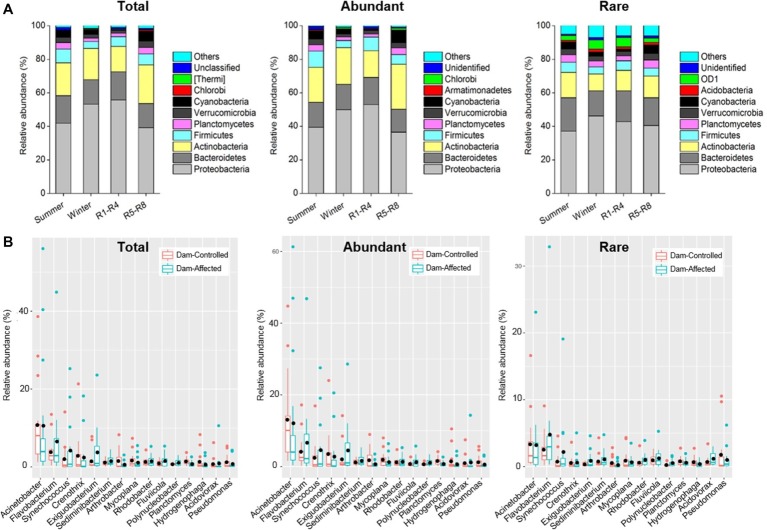
Relative abundances in dominant phyla **(A)** of the total, abundant, and rare bacterioplankton taxa in different sampling seasons and reservoir locations **(A)** and the differences in dominant genera between the dam-controlled and dam-affected sites **(B)**. In **(A)**, R1-R4 includes 12 sites in upstream reservoirs from Liyuan to Longkaikou, and R5-R8 includes 14 sites in downstream reservoirs from Ludila to Xiangjiaba in each sampling time. In **(B)**, box is drawn to represent values from lower 1/4 quantile to upper 1/4 quantile, medians are drawn as black bars within boxes. Total: whole bacterioplankton communities; Abundant: abundant taxa; Rare: rare taxa.

The dominant phyla across total samples were Proteobacteria, Bacteroidetes, Actinobacteria, Firmicutes, Verrucomicrobia, Planctomycetes, and Cyanobacteria, which together accounted for more than 90% of the total sequence data. The mean relative abundances of Proteobacteria and Actinobacteria in abundant taxa were higher than those for rare taxa, but Bacteroidetes exhibited higher relative abundance in rare taxa compared with the abundant ones (Supplementary Figure S5). Two-way ANOVA showed similar effects of sampling time and reservoir locations on the relative abundance of the dominant phyla for total and abundant taxa but different effects of these factors for rare taxa. Proteobacteria exhibited a significantly higher relative abundance in winter and R1-R4 samples for total and abundant taxa, but location had no significant effect on Proteobacteria for rare taxa (Figure 3 and Supplementary Table S4). The sampling location significantly affected the relative abundances of Planctomycetes and Cyanobacteria for the three groups of taxa (Supplementary Table S4), and these abundances were significantly higher in R5-R8 than in R1-R4 sites (Figure 3A). Sampling time did not significantly impact the relative abundance of Actinobacteria and Bacteroidetes for the abundant taxa, but their abundances were lower in winter than in summer for rare taxa (Figure 3A and Supplementary Table S4). Furthermore, the top 10 genera were similar for the total, abundant, and rare taxa. Acinetobacter was the most dominant genus, with mean relative abundances of 11.9, 13.9, and 3.76% for total, abundant, and rare taxa, respectively (Supplementary Figure S5). We compared the variation in the dominant genera between the dam-controlled and dam-affected sites. Notably, the relative abundances of genera *Flavobacterium*, *Synechococcus*, and *Exiguobacterium* were significantly higher in dam-affected sites than those in dam-controlled sites (Figure 3B).

### Environmental and Spatial Factors Correlated With Community Composition

Compared with R1-R4 sites, R5-R8 sites had significantly higher TN and NO_3_^−^ concentrations but lower TOC concentration (Supplementary Figure S7). The TN concentration was significantly higher, and the TOC concentration was lower in dam-affected sites than those in dam-controlled sites, and turbidity significantly decreased in dam-affected sites due to suspended matter sedimentation (Supplementary Figure S7). The influences of environmental and spatial factors on the community composition of different bacterioplankton taxa were next explored (Supplementary Table S5). Five environmental variables (water temperature, pH, DO, TOC, DSi) and one spatial variable (PCNM3) were significantly related to the community composition of the abundant taxa (*p* < 0.05). However, seven environmental variables (water temperature, pH, DO, TOC, DSi, TN, and NO_3_^−^) and two spatial variables (PCNM1 and PCNM3) exhibited significant effects on the variation of rare taxa (*p* < 0.05). Among the environmental variables, Dsi was most highly correlated with abundant taxa (*r* = 0.22, *p* < 0.001), while TOC was most highly correlated with rare taxa (*r* = 0.27, *p* < 0.001).

### Distance-Decay Relationship

**Figure 4:**
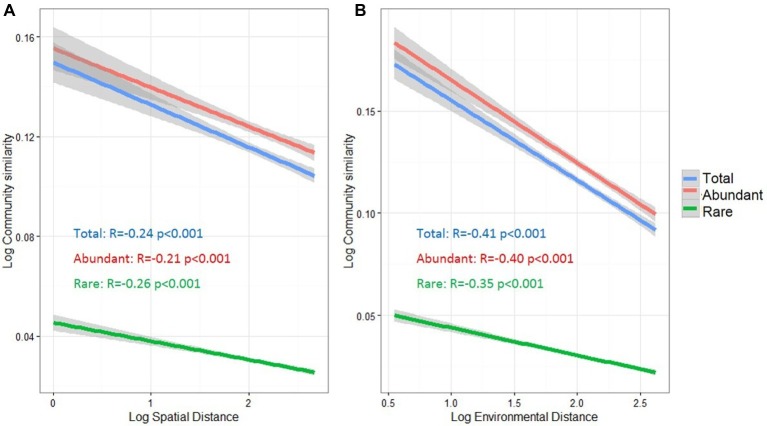
Distance-decay relationships between bacterioplankton community similarity, spatial distance **(A)** and environmental distance **(B)** in the cascade reservoirs of the Jinsha River. The spatial distance in meter, environmental dissimilarity based on Euclidean distance and community similarity based on Bray-Curtis distance was normalized using log10 (*x* + 1). Total: whole bacterioplankton communities; Abundant: abundant taxa; Rare: rare taxa.

To understand the potential relationships between microbial community similarity, spatial distance, and environmental heterogeneity, distance decay relationships were estimated for the total, abundant, and rare taxa (Figure 4). Community similarities of all three taxa groups were significantly decreased with increasing spatial and environmental distance (*p* < 0.001). Pearson’s correlation coefficients between microbial community similarity and spatial distance were −0.21 and −0.26 for abundant and rare taxa, respectively. For the distance decay relationship between community similarity and environmental distance, the abundant taxa had a higher correlation coefficient (*r* = −0.40) than that for rare taxa (*r* = −0.35).

### Relative Importance of Spatial and Environmental Processes

**Figure 5:**
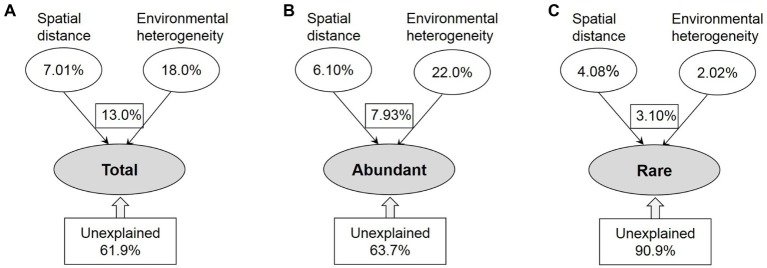
The variation partition analysis of the total **(A)**, abundant **(B)**, and rare **(C)** bacterioplankton taxa that can be explained by spatial distance and environmental heterogeneity. Total: whole bacterioplankton communities; Abundant: abundant taxa; Rare: rare taxa.

The contributions of spatial and environmental factors to variations in the total, abundant, and rare taxa were quantified by VPA (Figure 5). The amount of total variance that was explained by environmental and spatial factors was much lower for rare taxa (9.1%) than for the total (38.1%) and abundant (36.3%) taxa. Variation in the abundant taxa was primarily explained by environmental factor (22.0%); however, spatial factor (4.08%) explained more of the rare taxa variation than environment factor (2.02%). Remarkably, the spatial variables were more strongly correlated with rare taxa than with abundant ones, and the influence of environmental variables on bacterioplankton community assembly was stronger for abundant taxa than for rare taxa.

### Co-occurrence Networks of Different Taxa

**Figure 6:**
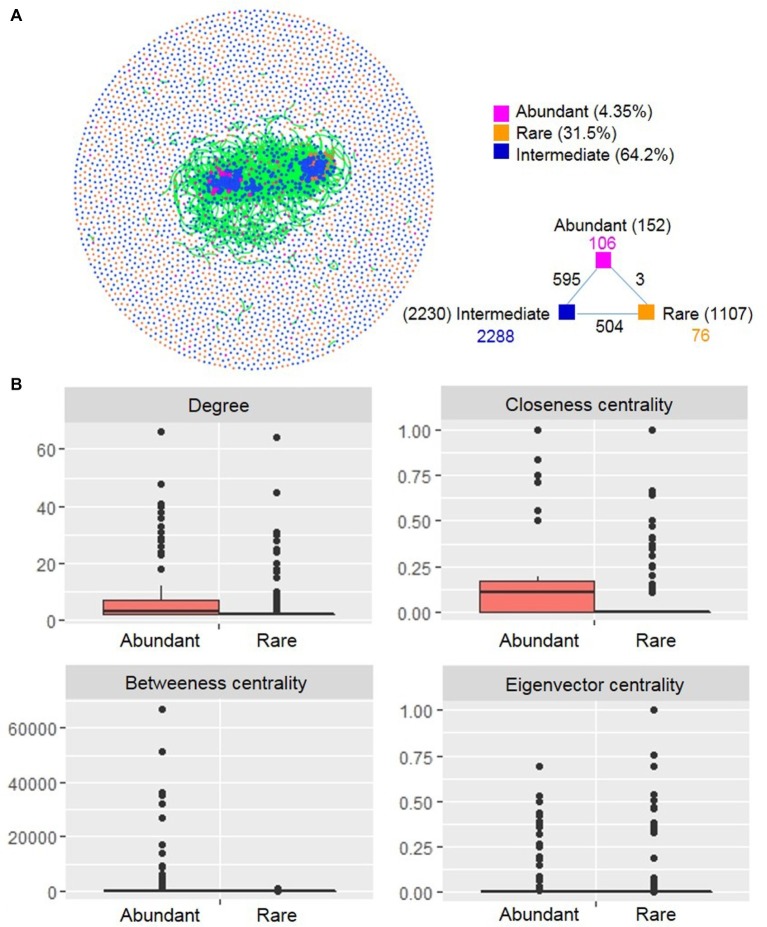
Co-occurrence network **(A)** and node-level topological features **(B)** of rare and abundant bacterioplankton taxa in the cascade reservoirs of the Jinsha River. In **(A)** each point represents an independent OTU, numbers inside parentheses represent the number of node, the black numbers represent external associations among each subcommunity, the numbers colored according to the categories represent the inner associations of each taxa. In **(B)**, box is drawn to represent values from lower 1/4 quantile to upper 1/4 quantile, medians are drawn as black bars within boxes; the values of degree, closeness centrality, and betweenness centrality were significantly different between rare and abundant taxa based on Student’s *t* test (*p* < 0.05). Abundant: abundant taxa; Rare: rare taxa.

A co-occurrence network was constructed based on correlation relationships (Figure 6A). The network included 3,572 associations (edges) between 3,498 OTUs (nodes), with a much higher proportion of positive correlations (99.5%) than negative ones (0.50%). Of the nodes, 152 and 1,107 belonged to abundant and rare taxa, respectively. In the global network, there were only three associations between rare and abundant nodes, numerous edges (595) between abundant and intermediate nodes, and 504 edges between rare and intermediate nodes. This result indicated that rare taxa seldom co-occurred with abundant taxa, but intermediate taxa frequently co-occurred with abundant and rare taxa. The degree distribution pattern of the global network was well fitted with the power-law model (*R*^2^ = 0.832) (Supplementary Figure S6), indicating a scale-free network structure and a non-random co-occurrence pattern. The network had modular structures due to their large modularity values (>0.5) (Supplementary Table S6).

We compared unique node-level topological features of abundant and rare taxa. The values of degree, closeness centrality, and betweenness centrality were all significantly higher for abundant taxa than for rare taxa (Figure 6B). We generated two subnetworks of abundant and rare taxa and compared their network-level topological features. The average degree, clustering coefficient, graph density, average path length, and diameter were all greater for the abundant taxa than for the rare taxa (Supplementary Table S6). A total of 22 OTUs were identified as keystone species and included Proteobacteria (8 OTUs), Planctomycetes (4 OTUs), Cyanobacteria (3 OTUs), Actinobacteria (2 OTUs), Nitrospirae (2 OTUs), Gemmatimonadetes (1 OTU), Bacteroidetes (1 OTU), and Armatimonadetes (1 OTU). Among these keystone species, half were considered abundant taxa and half were considered rare taxa (Supplementary Table S7).

## Discussion

### Diversity and Biogeographic Patterns of Abundant and Rare Taxa in Cascade Reservoirs

In this study, the rare taxa accounted for 76.1% of the OTUs of the total bacterioplankton community and had higher OTU richness and Pielou’s evenness than the abundant taxa in cascade reservoirs. This finding indicated that rare microbial species are important contributors to bacterioplankton species diversity (Galand et al., 2009; Liu et al., 2015a). Dam construction can alter river hydrological regimes and increase the water level in reservoirs, which will then change the water chemical properties and reduce habitat heterogeneity (Jordaan and Bezuidenhout, 2016; Timpe and Kaplan, 2017). In this study, the decreased alpha diversity of bacterioplankton due to damming may be attributed to the changes in water chemical properties (Supplementary Table S2 and Supplementary Figure S7), which may lead to the enrichment of adaptable species and the loss of sensitive species through a process of species sorting (Savio et al., 2015). Dam construction has a stronger impact on rare taxa than on abundant taxa (Figure 1), as rare species accounting for most of microbial diversity are less able to compete for nutrient resources and are more sensitive to environmental changes compared with abundant ones (Xue et al., 2018; Zhang et al., 2018). In addition, particle sinking and sedimentation due to damming have been shown to cause significant changes in suspended particle content and composition in river waters (Klaver et al., 2007; Ruiz-Gonzalez et al., 2013), which may in turn impact the diversity and composition of particle-associated microbial community (Dang and Lovell, 2016). Another explanation for the more significant loss of rare species by dam construction is that the sinking of suspended particles may have a stronger influence on the rare species than on the abundant species.

Cumulative impacts caused by multiple dams on a river and high river fragmentation have attracted considerable attention in recent years (Timpe and Kaplan, 2017). Several previous studies on riparian biota have found that multiple dams increased habitat fragmentation, exacerbated loss of primary vegetation, and reduced vegetation diversity relative to single dam systems (Jansson and Renofalt, 2000; Zhai et al., 2010; Li et al., 2012). In contrast, a study on low-head dams (<15 m in height) found no apparent cumulative effects along a river with multiple dams (Santucci et al., 2005). Until now, the cumulative effects of multiple large dams on bacterioplankton remain unclear. In this study, although the OTU richness of the rare taxa did not gradually decrease with the construction of additional dams from the upstream to the downstream (Supplementary Figure S3), relative change within specific area (from AH to LKK) did (Figure 1), reflecting the cumulative effect of the upstream cascade dams on rare species loss. However, the cumulative effect on rare species loss did not stably retain in the downstream reservoirs (from LDD to XJB), particularly for GYY and XJB (Figure 1). This result may be attributed to the longer river distances between two adjacent dams and the larger catchment areas in the downstream reservoirs than in the upstream ones (Supplementary Table S1). Flowing downriver, with increasing river distance, facilitates the increase of newly introduced allochthonous bacterial species from riparian soils or tributaries to the river community (Read et al., 2015; Savio et al., 2015), probably leading to relatively less species loss in the downstream reservoirs. In addition, compared with the upstream four dams with shorter separation distance, the longer distance between two dams in downstream may favor the self-recovery of bacterioplankton diversity in the river ecosystem (Wang et al., 2018). This may also explain the weaker effects of dam construction on rare species loss in downstream reservoirs. Further studies are needed to elucidate the potential self-recovery ability of bacterioplankton communities in highly regulated rivers and provide scientific evidence to management plans and policies for cascade hydropower development in the future.

Recent studies on the biogeography of microbial communities in some large rivers have reported distinct bacterial assemblages for the upstream and downstream sites (Chen et al., 2018; Wang et al., 2018). Additionally, Liu et al. (2018) observed that seasonal differences in bacterioplankton communities were significant in the Yangtze River, China. Given the large spatial and environmental variations of our samples, it would be expected that reservoir location and environmental factors could greatly affect the spatial or temporal variability of bacterioplankton community composition (beta diversity). The abundant taxa showed significantly lower beta diversity than the rare taxa (Figure 2), indicating that the abundant taxa may be the most common taxa in the various sampling environments (Nolte et al., 2010; Xue et al., 2018). Nevertheless, the rare taxa exhibited a biogeographic pattern similar to that of abundant ones (Figure 2). This similarity in the biogeographic patterns implied that rare bacteria biosphere was not a random collection of taxa (Galand et al., 2009) and rare taxa might respond to environmental changes in a similar manner to abundant ones (Liao et al., 2017; Zhang et al., 2018). Differently, a previous study carried out in an activated sludge bioreactor demonstrated clear differences in temporal dynamic patterns for the abundant and rare taxa (Kim et al., 2013). This discrepancy may be attributed to different bacterial assembly patterns between natural aquatic ecosystems and artificial systems.

According to the taxonomic analysis, the dominant phyla of the abundant and rare taxa were similar, regardless of reservoir location and sampling season, and the top three phyla (Proteobacteria, Bacteroidetes, and Actinobacteria) are typical freshwater bacteria in rivers (Fortunato et al., 2013; Wang et al., 2017, 2018). Environmental gradient in temperature has been reported to strongly affect the temporal variability of bacterioplankton assemblages (Liu et al., 2018). In this study, Actinobacteria and Bacteroidetes for rare taxa were more prevalent in winter than in summer (Figure 3A). It would be expected that the significant change in water temperature from winter (average 14.8°C) to summer (average 19.4°C) in the Jinsha River appears to have influenced the community composition of rare taxa. Previous research studies of the Thames River (Read et al., 2015), the Danube River (Savio et al., 2015), and the Lancang River (Wang et al., 2018) reported a transition from a Bacteroidetes- to an Actinobacteria-dominated community from upstream to downstream sites. In this study, for the total and abundant taxa, the downstream reservoirs exhibited higher relative abundances of Actinobacteria compared with the upstream reservoirs but lower relative abundances of Proteobacteria and Bacteroidetes (Figure 3A). Such transition in dominated bacterial phyla has been proposed to mainly depend on the adaptation of individual bacterial species to diverse environmental conditions (Fierer et al., 2011; Read et al., 2015). The upstream reservoirs of the Jinsha River, with a low level of competition, would favor rapidly growing species that can utilize available resources quickly (r-strategists like Bacteroidetes) (Weinbauer and Hofle, 1998). However, due to water transitions downstream and disturbance caused by multiple dams, the competition may become more intense, leading to dominance of k-strategist species (e.g., Actinobacteria) that are more competitive and have lower growth rates and narrower niches (Simek et al., 2006).

Notably, the Cyanobacteria in both abundant and rare taxa were stimulated in the downstream reservoirs (Supplementary Figure S5). We also found enrichment of *Synechococcus*, a genus belonging to Cyanobacteria, in abundant and rare taxa due to dam construction (Figure 3B). According to our measurements, the relatively higher TN concentration in downstream reservoirs and dam-affected sites (Supplementary Figure S7) may stimulate an increase in cyanobacterial species, as it has been reported that the abundance of Cyanobacteria was positively correlated with N nutrient content in waterbody (Gkelis et al., 2014). Additionally, the longer retention of riverine water may also favor the enrichment of Cyanobacteria in dam-formed reservoirs (Ruiz-Gonzalez et al., 2013). Furthermore, the *Flavobacterium* belonging to the Flavobacteriia class in the Bacteroidetes phylum, which functions to remineralize large components of phytoplankton organic matter using its highly efficient extracellular system (Buchan et al., 2014), was enriched in dam-affected sites. Phytoplankton blooms have been demonstrated to sustain active and diverse bloom-associated bacterial populations, especially Flavobacteriia (Alonso et al., 2007). In consequence, more attention should be paid to the elucidation of bacteria-phytoplankton interactions in reservoirs.

### Controlling Factors for Biogeographic Distributions of Abundant and Rare Taxa

Environmental (deterministic) and dispersal-related (stochastic) processes are two primary processes determining the biogeographical distributions of microbial communities (Jiang et al., 2018; Zhang et al., 2018). Distance decay relationships can be used to evaluate the importance of these two ecological processes, since environmental selection and dispersal limitation can greatly affect the pattern of distance decay (Xiao et al., 2018). In this study, we observed that bacterioplankton, regardless of community taxa, significantly followed spatial and environmental distance-decay relationships (Figure 4), suggesting that dispersal-related and environmental processes might act concurrently to regulate bacterioplankton assemblages.

Notably, the spatial distance-decay relationship of abundant taxa (*r* = −0.21) was weaker than that of the rare ones (*r* = −0.26) (Figure 4), indicating that abundant taxa might have a weaker dispersal limitation for these studied reservoirs. The different responses of abundant or rare taxa to dispersal limitation may be explained from two aspects. First, more abundant taxa were found in multiple sites (Supplementary Figure S4), demonstrating that abundant bacteria with high abundance have a decreased probability of extinction and an increased probability of dispersal (Liao et al., 2017), thereby resulting in widespread or ubiquitous distribution in the cascade reservoirs. Second, abundant bacteria utilize a wider spectrum of resources than rare bacteria, potentially allowing a more cosmopolitan distribution (Hambright et al., 2015), whereas the rare taxa are more likely to show a restricted distribution (Pedrós-Alió, 2006; Logares et al., 2014). However, Pedrós-Alió (2012) speculated that rare taxa have potentially unlimited dispersal capacity due to their low loss rates. Overall, the dispersal capacities of different taxa remain poorly understood and may depend on the spatial scale of research area, habitat type, current movement, and water masses (Logares et al., 2014; Mo et al., 2018; Zhang et al., 2018). For example, Liao et al. (2017) reported the significantly weak distance-decay relationship of both rare and abundant taxa in freshwater lakes on Yungui Plateau, China, which differs with our observation of spatial distance decay (Figure 4). This difference might be attributed to their less sampling sites (only 21 sites), compared to that of our study, and the spatial separation of cascade dams in this study. Clearly, further investigation is required to characterize the different dispersal potential for abundant and rare taxa.

Moreover, bacteria attached to suspended particles may constitute as much as 90% of total bacterioplankton production in riverine systems (Crump and Baross, 2000). Dam construction could obviously cause species loss in river waters due to sedimentation of suspended particles colonized by bacteria to the bottom of reservoirs (Ruiz-Gonzalez et al., 2013). Thus, species loss may also contribute to drive the biogeographical distributions of bacterioplankton communities in a dammed river. Compared with the abundant taxa, the rare taxa may be more easily affected by particle sinking-induced species loss, because the rare taxa that represented by only a few individuals with very low abundance may have high loss rate and are likely to be most at risk of extinction (Pedrós-Alió, 2006; Liao et al., 2017). On the other hand, the differences in the relative abundance of dominant taxonomic groups between abundant and rare taxa may also explain the stronger effects of particle sinking on rare species loss due to dam construction. Free-living and particle-associated bacteria are known to differ phylogenetically across ecosystems (DeLong et al., 1993; Besemer et al., 2005). For example, Alphaproteobacteria and Actinobacteria are dominant free-living lifestyle, whereas Gammaproteobacteria and Betaproteobacteria have often been found associated to particles (DeLong et al., 1993; BÖckelmann et al., 2000). It is obvious that the particle-associated bacterial groups (Gammaproteobacteria and Betaproteobacteria) were more abundant in the rare taxa than in the abundant ones in our study (Supplementary Figure S5), probably accounting for the rare species loss due to particle sinking in the cascade reservoirs.

The importance of environmental selection suggests that most species in abundant and rare taxa are not ecologically equivalent but instead have strict requirements for environmental condition (Liao et al., 2017). Interestingly, the environmental variables that constrained abundant and rare taxa are not exactly the same (Jiao et al., 2017; Mo et al., 2018). We found that environmental factors including water temperature, pH, DO, TOC, and DSi were significantly related to variations in the abundant and rare taxa (Supplementary Table S5). A previous study on the Yangtze River concluded that water temperature and DO strongly influenced the spatial distribution of indigenous bacterioplankon communities (Liu et al., 2018). Nutrient concentrations (e.g., TOC and DSi) were also suggested as important environmental factors affecting bacterial community composition, as they are essential for microbial growth and development (Wang et al., 2015b). However, TN and NO_3_^−^ significantly affected rare taxa but not the abundant taxa (Supplementary Table S5). One explanation for this result is that abundant and rare bacterial taxa might play different functions and have different ecological niches in aquatic ecosystems (Mo et al., 2018; Zhang et al., 2018). An alternate explanation is that most abundant species may have sufficient intercellular storage of nitrogen, making them less sensitive to changes of nitrogen concentration in a waterbody (Liao et al., 2017).

Although microbial communities are normally assembled by both dispersal-related process and environmental variables, quantifying the relative contributions of these processes remains an important challenge of microbial ecology (Liao et al., 2017; Mo et al., 2018). VPA is a good tool to compare the relative importance of spatial and environmental factors (Lindstrom and Langenheder, 2012; Xiao et al., 2018). Based on VPA, we found the dominance of environmental factors for abundant taxa (Figure 5), which might be explained by the intense selective strength of habitat conditions in our study area and the relatively high environmental heterogeneity between upstream and downstream reservoirs (Supplementary Figure S7). Wang et al. (2013) suggested that the impact of environmental selection could overwhelm that of dispersal-related process when the selective strength of local habitat conditions exceeds a conceptual threshold, especially for the systems along large environmental gradients. In addition, the proportion of generalists detected among abundant taxa may affect the relative importance of environmental and spatial processes, as the assembly of generalists was strongly governed by spatial process (Wang et al., 2013; Liao et al., 2016). Liao et al. (2017) found that environmental selection exhibited a greater influence on abundant taxa than spatial process in lakes of Yungui Plateau, China, because of the lower proportion of environmental generalists detected in abundant taxa. In this study, however, most abundant bacteria appeared to be habitat generalists, given 97.3% of abundant OTUs occupied >50% of sites (Supplementary Figure S4). Most likely, additive effects of multiple environmental factors or more complex mechanisms may generate and maintain the structure of an abundant biosphere in the cascade reservoirs of a large river. Different from abundant taxa, rare taxa had a slightly stronger response to spatial factor (Figure 5), probably because with higher diversity, these taxa might occupy greater varieties of ecological niches (Liao et al., 2016; Jiao et al., 2017). In addition, the unexplained variation of rare taxa was significantly higher than that of the entire and abundant communities (Figure 5). Thus, another reason for the dominant influence of spatial process on rare taxa is that other additional biotic and abiotic factors those are related to the biogeography of rare taxa were not measured in this study.

### Co-occurrence Patterns of Abundant and Rare Taxa

Co-occurrence network analysis can reveal microbial interactions, keystone species, and ecological assembly mechanisms that cannot easily be identified by conventional studies of microbial richness and composition (He et al., 2017). In addition to explore the influence of dispersal limitation and environmental selection, we next evaluated the potential contribution of microbial interactions to their ecological assembly by correlation-based network topological characteristics analysis. The generated network of the entire community exhibited structural characteristics similar to those observed in other ecosystems (He et al., 2017; Tian et al., 2018), including the power-law distribution, non-randomly connected property, and modular structure. Within the network, rare taxa rarely co-occurred with abundant taxa, which may be partly because that the rare taxa were detected in only few samples (Supplementary Figure S4) and were therefore statistically less likely to interact with other taxa.

The topological features can reflect the centrality and interaction of microbial species in a network (Deng et al., 2012). For example, the degree (the number of adjacent edges) describes the level of connectedness between OTUs, and betweenness centrality (the number of shortest paths going through a node) can be used to assess the centrality of each OTU in the network (Ma et al., 2016). The topological feature values of the abundant taxa subnetwork were higher than that for the rare taxa (Supplementary Table S6). This substantial influence of abundant taxa in the co-occurrence network is probably attributed to their ubiquitous distribution in our sampling sites and implies potential strong effects of environmental changes on particular species in cascade reservoirs, as described previously (Jiao et al., 2017). For instance, three keystone species of abundant taxa identified in this study belong to Cyanobacteria (Supplementary Table S7), a phylum that is widely reported to be more abundant in dam-formed reservoirs due to the increased nutrient concentration (Ruiz-Gonzalez et al., 2013; Wang et al., 2018; Chen et al., 2019). In contrast, Xue et al. (2018) reported that rare planktonic eukaryotes play more important roles in network persistence than abundant taxa in a subtropical reservoir following a cyanobacterial bloom event. These different results may reflect differences in the studied plankton communities (planktonic eukaryotes vs. bacterioplankton) or differences in the strength of ecological selection. In addition, less-abundant taxa can act as important keystone taxa in a microbial network (Xue et al., 2018). In this study, half of the keystone species were rare taxa, suggesting a potential role of rare species to maintain the structure and stability of microbial communities. Within the rare taxa, keystone species belonged to the genera *Nitrospirae* and *Pseudomonas* (Supplementary Table S7), known for their great contributions to ecosystem nitrogen cycling at relatively low abundance (Huang and Tseng, 2001). Rare microbes must sustain a vast functional gene pool and can indirectly enhance ecological function of the abundant microbial biosphere (Jousset et al., 2017).

## Conclusion

Our results clearly demonstrated that river damming reduced the alpha diversity of bacterioplankton community in reservoir water, especially for rare taxa. The community compositions of both abundant and rare taxa were significantly distinct in different sampling seasons (summer vs. winter) and different reservoir locations (upstream reservoir vs. downstream reservoir). Dispersal limitation and environmental selection simultaneously affected the community assembly of bacterioplankton. The abundant sub-community was primarily impacted by environmental selection, while spatial factor exhibited a slightly greater influence on rare sub-community compared to environmental factor. Co-occurrence network analysis showed that the bacterioplankton network had non-random occurrence property and modular structure. Abundant taxa with closer relationship may play more important roles in maintaining the stability and persistence of a microbial network than rare taxa. Based on these results, we concluded that the observed similar biogeographic pattern of abundant and rare taxa was due to distinct assembly mechanisms in cascade reservoirs. Our results expand our knowledge of the biogeographic patterns of bacterioplankton and provide a new perspective for the ecological significance of abundant and rare microbes in highly regulated rivers. However, this study only focused on the general changes of entire bacterioplankton community, a major aspect missing from this study is information on the functional responses of particle-associated and free-living bacterial communities to damming. Further research on the impacts of cascade dams on functional traits of riverine particle-associated and free-living bacterial communities will provide a better understanding of the potential ecological consequences of dam construction and river regulation.

## Data Availability Statement

Publicly available datasets were analyzed in this study. This data can be found in the NCBI repository: SRP219313, SRP219317.

## Author Contributions

JC, PW, and CW designed the study. JC, XW, LM, QY, and SL performed the field sampling and the experiment. JC and SS analyzed the data and wrote the manuscript.

### Conflict of Interest

The authors declare that the research was conducted in the absence of any commercial or financial relationships that could be construed as a potential conflict of interest.

## Footnotes

^1^http://greengenes.lbl.gov/
